# 3D-assisted corrective osteotomies of the distal radius: a comparison of pre-contoured conventional implants versus patient-specific implants

**DOI:** 10.1007/s00068-023-02415-5

**Published:** 2024-01-23

**Authors:** Miriam G. E. Oldhoff, Nick Assink, Joep Kraeima, Jean-Paul P. M. de Vries, Kaj ten Duis, Anne M. L. Meesters, Frank F. A. IJpma

**Affiliations:** 1 Department of Trauma Surgery, University of Groningen, University Medical Center Groningen, Groningen, The Netherlands; 2 3D Lab, University of Groningen, University Medical Center Groningen, Groningen, The Netherlands; 3 Department of Oral and Maxillofacial Surgery, University of Groningen, University Medical Center Groningen, Groningen, The Netherlands; 4 Department of Surgery, University of Groningen, University Medical Center Groningen, Groningen, The Netherlands

**Keywords:** Corrective osteotomy, Distal radius, Virtual surgical planning, 3D technology, Patient-specific implants, Guided surgery

## Abstract

**Purpose:**

There is a debate whether corrective osteotomies of the distal radius should be performed using a 3D work-up with pre-contoured conventional implants (i.e., of-the-shelf) or patient-specific implants (i.e., custom-made). This study aims to assess the postoperative accuracy of 3D-assisted correction osteotomy of the distal radius using either implant.

**Methods:**

Twenty corrective osteotomies of the distal radius were planned using 3D technologies and performed on Thiel embalmed human cadavers. Our workflow consisted of virtual surgical planning and 3D printed guides for osteotomy and repositioning. Subsequently, left radii were fixated with patient-specific implants, and right radii were fixated with pre-contoured conventional implants. The accuracy of the corrections was assessed through measurement of rotation, dorsal and radial angulation and translations with postoperative CT scans in comparison to their preoperative virtual plan.

**Results:**

Twenty corrective osteotomies were executed according to their plan. The median differences between the preoperative plan and postoperative results were 2.6° (IQR: 1.6–3.9°) for rotation, 1.4° (IQR: 0.6–2.9°) for dorsal angulation, 4.7° (IQR: 2.9–5.7°) for radial angulation, and 2.4 mm (IQR: 1.3–2.9 mm) for translation of the distal radius, thus sufficient for application in clinical practice. There was no significant difference in accuracy of correction when comparing pre-contoured conventional implants with patient-specific implants.

**Conclusion:**

3D-assisted corrective osteotomy of the distal radius with either pre-contoured conventional implants or patient-specific implants results in accurate corrections. The choice of implant type should not solely depend on accuracy of the correction, but also be based on other considerations like the availability of resources and the preoperative assessment of implant fitting.

## Introduction

Distal radius fractures are common and account for 12% of all injury-related emergency room visits in the Netherlands in 2021 [1]. Only 13% of distal radial fractures are treated with open reduction and internal fixation [2]. The majority of fractures is treated non-operatively with or without closed fracture reduction and cast immobilization [2]. Unfortunately, a malunion occurs in approximately 5% of non-surgically treated patients after secondary fracture displacement in a cast. This can lead to pain, restricted range of motion, and early onset of osteoarthritis [3]. Depending on the severity of the malunion, a corrective osteotomy may be required. To enhance surgical outcomes, three-dimensional (3D) techniques have been introduced to create patient-specific virtual surgical models and 3D printed guides, facilitating the translation of the virtual surgical plan to the operating room. These 3D techniques have demonstrated promising results in terms of postoperative accuracy of osteotomies, functional outcomes, and reduced operating times [4–6]. A recent literature review indicates a positive trend toward the use of 3D techniques compared to the use of merely 2D imaging modalities (e.g., without virtual surgical planning, 3D printed models and guides) [7–9]; however, many large randomized control trials have not been conducted yet.

Current verified 3D work-ups consist of the use of 3D printed saw-, drilling-, and reposition guides in combination with a pre-contoured conventional (i.e., of-the-shelf) implant or patient-specific (i.e., custom-made) implant. Several of these conventional commercially available implants are used for fixation of the distal radius, so-called anatomical osteosynthesis plates. Due to the abnormal bone anatomy caused by the malunion, the conventional implants might not fit, which can lead to suboptimal plate-to-bone contact. Moreover, this increased distance between the plate and the bone can lead to additional soft tissue irritation. Manual pre-contouring [10–12] of the plate is possible to optimize the shape and ensure proper fit. Yet this pre-contouring is moderately preferred, since this can change the mechanical integrity of the implant [6] and functionality of the screw holes. Then the question arises if sufficient pre-contouring is even possible to perform accurate corrective osteotomies with proper implant fitting. The risk of improper fit is further amplified when the implants are used for corrective osteotomy due to the distorted anatomy caused by malunion and potential excess bone growth. Rosseels et al. [11] demonstrated an under-correction compared to the virtual plan when corrective osteotomies of the forearm were performed with commercially available implants combined with surgical guides. Similarly, Oka et al. [10] stated that a mismatch between the shape of an off-the-shelf volar plate and malunited distal radius can result in under-correction. The possible over- or under-correction of 3D-assisted corrective surgeries with non-fitting conventional implants [10–12] can result in a suboptimal functional outcome. Therefore, alternatives to these conventional implants need to be considered.

Patient-specific implants (i.e., custom-made) are designed to fit the patients’ bone anatomy and have the ability to provide optimal screw locations and trajectories. They have full plate-to-bone contact at the final preferred bone position, based on the 3D virtual models created by the patients’ computed tomography (CT) data. In our clinic, corrective limb osteotomies are currently performed using a 3D-assisted two-step approach, resulting in reliable, feasible, and accurate results [13]. Corrective osteotomies of the radius with both a patient-specific implant and a surgical guide showed high accuracy and reproducibly of positioning on artificial bone [14, 15], in cadaveric studies [16] and in clinical reports [17–19]. Both techniques present distinct advantages and disadvantages. With regard to the preoperative process, conventional implants are readily available with all the necessary legal and technical documentation and approvals. However, the entire workflow involves additional steps to accommodate pre-contouring, and achieving a perfect fit to the bone is often challenging. On the other hand, patient-specific implants offer a more streamlined workflow and may provide a superior fit to the patient’s anatomy. Nevertheless, their adoption requires significant investments in terms of expertise and time to ensure compliance with legal and technical requirements and to train personnel adequately. However, a direct comparison of the postoperative results of the personalized two-step approach using either a patient-specific or a conventional pre-contoured implant has not been investigated. Therefore, the objective of this study was to assess the accuracy of the 3D-assisted corrective osteotomy using either a pre-contoured conventional (i.e., of-the-shelf) implant or a patient-specific (i.e., custom-made) implant. We assessed the feasibility of both techniques, and investigated the postoperative accuracy of the planned corrective osteotomy and implant placement using 20 cadaveric upper extremities.

## Materials and methods

### Specimens

A total of ten full-body Thiel embalmed human cadaver specimens from the anatomy department were used [20]. Each specimen underwent similar corrective osteotomy surgery on both distal radii, with the left radius fixated using a patient-specific implant and the right radius fixated using a conventional implant. CT data of the upper extremity were acquired that included the entire radius according to our standard clinical imaging protocol (0.6 mm slice thickness, voxel size 0.4 mm), which was used as base for our 3D planning.

### 3D surgical planning

**Fig. 1:**
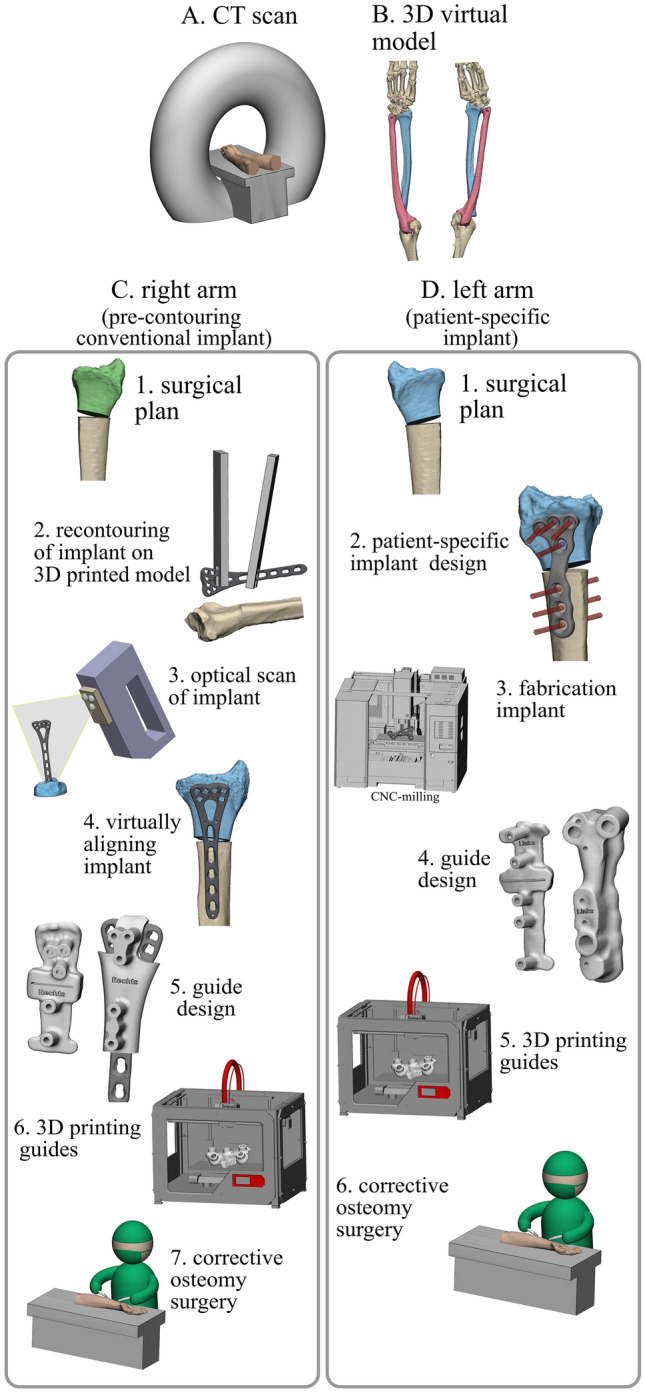
3D-assisted workflow for management of corrective osteotomies of the distal radius. The workflow illustrates both a pre-contoured conventional implant (**c**) and the use of the patient-specific implant (**d**) in combination with surgical guides

The first step involved segmentation of the CT data using Mimics Medical software (version 23.0, Materialise, Leuven, Belgium) to create 3D models of the entire left and right radius. A pre-set threshold value for bone (≥ 226 Hounsfield Units) was applied to select the bone region. The radius was then separated from the surrounding bones using split mask tool and edit mask tool (Fig. 1b). The virtual 3D models were then imported in the 3D software 3-Matic (version 17.0, Materialise, Leuven, Belgium). The human cadaveric specimens had no deformities; thus, ‘normal’ corrective osteotomies (e.g., turning malunions to normal anatomy) were not possible. Therefore, an alternative test setup with corrective osteotomies in a reversed direction (e.g., turning normal anatomy to malposition) was used to assess our 3D-assisted workflow with pre-contoured conventional implants versus patient-specific implants. Malpositions of the distal radius were created by performing a virtual osteotomy and turning the distal part in multiple directions (e.g., rotation, dorsal angle/tilt and radial angle/inclination) (Fig. 1c1/d1). The planned reversed corrections (defined in three terms of angulation and translation of the center of mass) varied in degree of malposition per case and are shown in Appendix 1. Case 1–5 underwent relatively small corrections, while case 6–10 underwent larger corrections.

### Right arms: pre-contouring of the conventional implant

The process of the right arm started by selecting the proper implant. All corrections in this group were fixated using a conventional 2.4 mm Variable Angle LCP Two-Column Volar Distal Radius Plate (DePuy Synthes, Raynham, MA, USA) with a length of either 66 mm or 75 mm. The complete 3D surgical plan for the conventional implant is depicted in Fig. 1c. The virtually planned reversed corrective osteotomy was 3D printed as one solid model out of polyamide powder using selective laser sintering techniques (SLS). The conventional implant was manually pre-contoured using this physical model as a reference (Fig. 1c2). The pre-contoured implant was then scanned optically with an Artec Space Spider (Artec 3D, Luxembourg, Luxembourg) (Fig. 1c3). Subsequently, the image file of the scanned pre-contoured conventional implant was imported into the virtual surgical plan and used as starting point for the guide design (Fig. 1c4).

### Left arms: patient-specific implant

**Fig. 2:**
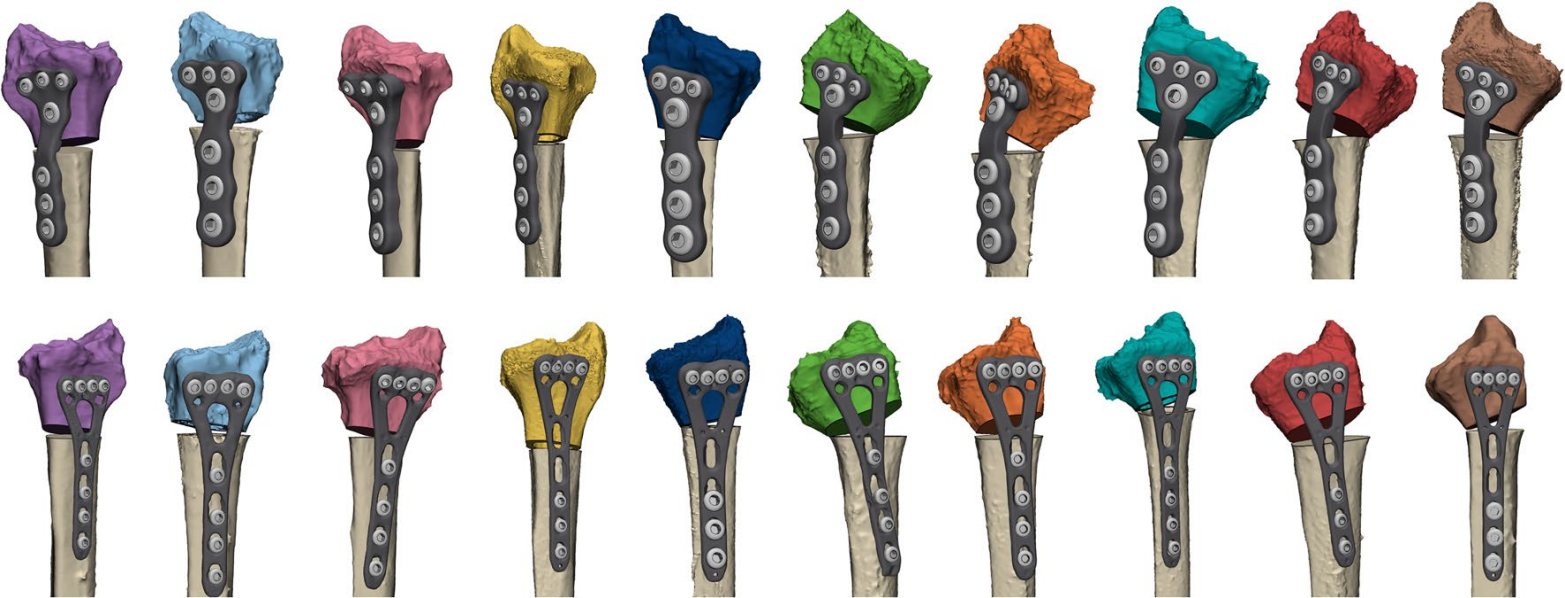
All 20 virtually planned corrective osteotomies with the implant and screws. Every color illustrates a case. The top row pictures demonstrate the left radii with the patient-specific implants, the bottom row shows the right radii with the manually pre-contoured conventional implants. All ten patient-specific implants were designed with three distal screw holes that fit 2.4 mm cortical screws heads, along with one proximal screw hole for a 3.5 mm cortical screw head distal from the osteotomy. Three screw holes for 3.5 mm cortical screwheads were placed proximal of the osteotomy

The virtual surgical plans for performing reversed corrective osteotomies of the left radius included the use of patient-specific implants. These implants were designed on the desired end-position of the distal radius (Fig. 1d1). The preferred screw trajectories in relation with the planned osteotomy were first pre-determined, followed by the design of the implant using 3-Matic software (version 17.0, Materialise, Leuven, Belgium), Solidworks Professional software version 2020 (Dassault Systèmes Solidworks), and the Geomagic package for Solidworks (3D Systems) (Fig. 1d2). The patient-specific implants were designed within 1 day and were in line with previously clinically tested custom-made osteosyntheses plates from our group [21, 22]. All ten patient-specific implants and ten pre-contoured implants are illustrated in Fig. 2.

The 3D virtual surgical plan, including screw trajectories and plate design, was discussed in a multidisciplinary meeting involving trauma surgeons, technical physicians, and an engineer for each case. The patient-specific implants were manufactured out of a medical grade titanium alloy by a 5-axis milling machine at a regional medical company Witec Medical B.V. (Stadskanaal, the Netherlands) within 3 days.

### Surgical guides

Two surgical guides were designed for each implant, according to our two-step approach for 3D-guided patient-specific corrective osteotomies [13] (Fig. 1c5 *conventional implant*/Fig. 1d4 *patient-specific implant*). The first guide, the osteotomy guide, was used to create the osteotomy at the desired location and to place the k-wires. Therefore, it included holes for drilling and placing all the k-wires, along with a slot for cutting the osteotomy. The second guide, the reposition guide, was designed to direct the bones toward their planned position by aligning the k-wires in a parallel manner. After removal of the first guide, this second guide was placed over the k-wires and forced the bone in the planned position. The guides used for the patient-specific implants included multiple cylinders in which a stainless-steel drill sleeve could be inserted to guide the drill and place the screws in the pre-planned direction. These drill sleeves were milled from stainless-steel 316 L. All surgical guides were designed in 3-Matic Medical (version 17.0, Materialise, Leuven, Belgium). They were 3D printed using medical-certified polyamide powder and selective laser sintering techniques (Fig. 1c6/d5).

### Surgical procedure

**Fig. 3:**
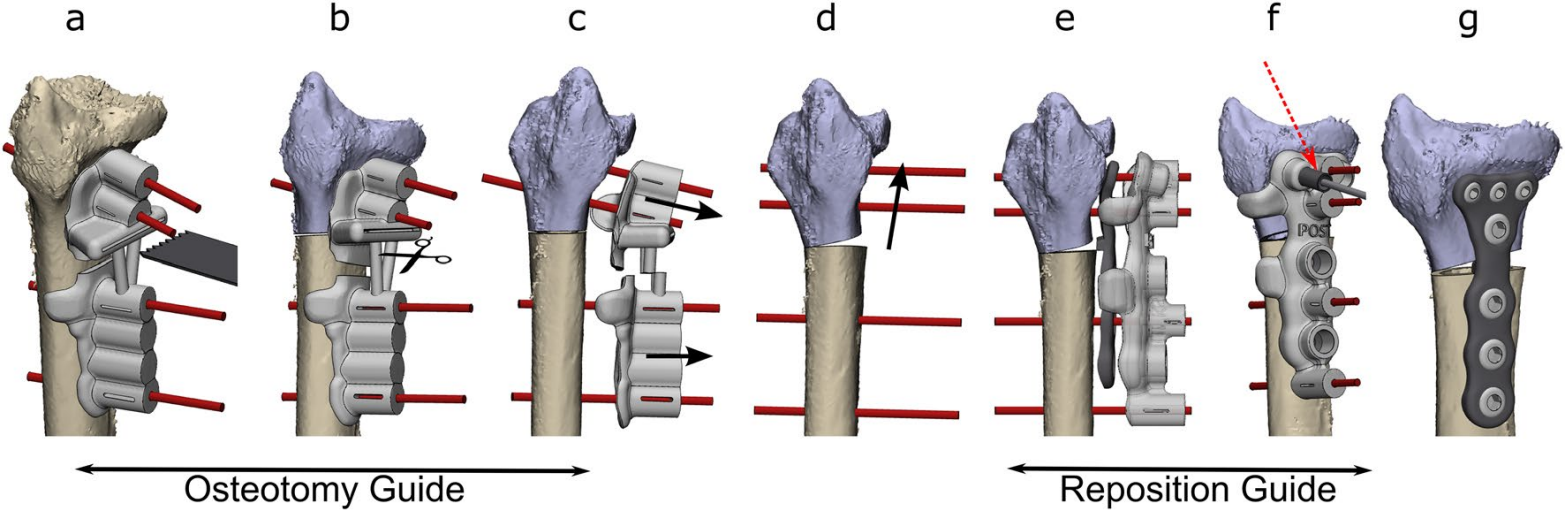
Schematic workflow of the two-step approach for 3D-guided patient-specific corrective osteotomies. The osteotomy guide was placed on the radius and four k-wires (red) were placed through the guide. The guide also had a slot for the saw for the osteotomy (**a**). The osteotomy guide was cut in two (**b**) and removed separately (**c**). The k-wires were moved parallelly, rotating and translating the distal bone to the planned position (**d**). The reposition guide and implant were placed on top of each other over the parallel k-wires (**e**). Several screws were pre-drilled using drilling sleeves (dark grey, see red arrow) (**f**). The guide was removed and the final screws were placed to fixate the implant (**g**). The location and direction of the k-wires were determined on the virtual surgical plan. Two k-wires were placed on each side of the osteotomy through the screw holes of the plate in the parallel manner, to allow for easy placement of the reposition guide. The two distal k-wires were then translated and rotated exactly as much as the distal bone part was for the corrective osteotomy, thus moving them in the right position for the first guide

The modified Henry approach was performed on each cadaver by an experienced trauma surgeon (FIJ). The radius was exposed according to the standard of care. After this, the osteotomy guide was positioned on the bone and the k-wires were drilled through the designated holes. The osteotomy was then performed using the defined slot (Fig. 3a). Afterwards, the guide was cut in half (Fig. 3b), facilitating removal (Fig. 3c). The k-wires were then repositioned in a parallel manner (Fig. 3d). The implant and the second guide, known as the reposition guide, were placed on top of each other and used to enclose the implant and ensure the bone maintained the planned position (Fig. 3e). The reposition guide included slots for the drill sleeves, which were used to drill and place the screws in the pre-planned directions (Fig. 3f). Subsequently, the reposition guide was removed and the remaining screws were placed (Fig. 3g). Finally, the implants’ position, achieved correction and screw positions were verified with intraoperative fluoroscopy.

### Postoperative evaluations

**Fig. 4:**
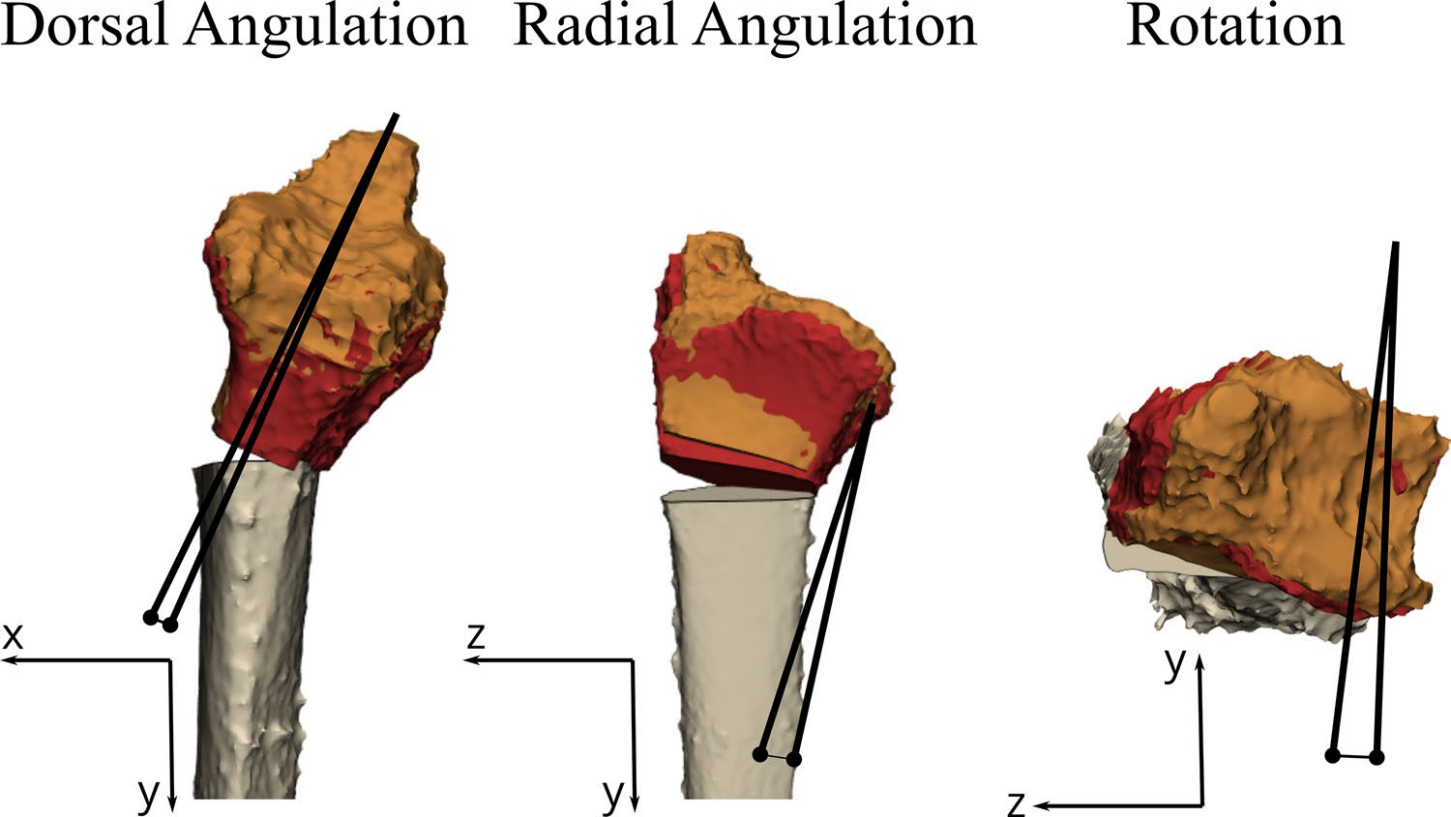
Illustration of the 3D measurements in the three planes; rotation, dorsal angulation, and radial angulation between the preoperative 3D virtual surgical plan (orange) and the postoperative achieved position (red). The angles were measured on the coordinate system of a plane representing the original sagittal plane of the preoperative radius, which was manually created in 3-Matic. The dorsal angulation, radial angulation, and rotation were measured in the *x*–*y* plane, *y*–*z* plane, and *x*–*z* plane, respectively. From left to right: sagittal view, coronal view, and axial view

A postoperative CT scan (0.6 mm slice thickness; iterative metal artefact reduction) was made from each specimen to evaluate the postoperative results. This CT scan was used to make a postoperative 3D model of the radius with the plate and screws. To assess the accuracy of the postoperative alignment, the distal bone part was duplicated and aligned with the postoperative model. Then the positions of the distal parts of the radius from the virtual plan were compared to those obtained from the postoperative CT scan. Measurements were performed in 3D, encompassing rotation, dorsal angulation, and radial angulation, to evaluate the alignment between the preoperative 3D virtual plan and the postoperative result (Fig. 4). In addition, translation was determined by measuring the Euclidean distance in millimeters between the center of mass of the planned distal radius bone part and the aligned postoperative part.

The screw directions of the patient-specific implant were virtually planned during the preoperative planning phase. In each case, three to four screw trajectories were pre-drilled using the reposition guide. The differences in screw direction for these screws were assessed by comparing the planned and postoperative screw trajectories, through matching of the postoperative plate with the planned plate. The difference in degrees in screw direction was measured in 3D between the inertia axes of the virtually planned and postoperative screw trajectories. Finally, the postoperative position of the implant was compared to the preoperative planned position. The Euclidean distance in millimeters between the center of mass of the implant was measured and taken as the translation of the implant.

### Statistical analysis

Statistical analysis was performed using SPSS (version 23, IBM, Chicago, IL, US). Continuous variables for the achieved accuracy were presented as median and interquartile range (IQR) for the overall group and separately for the patient-specific implant group and the pre-contoured conventional group. Here the achieved accuracy was taken as the differences between the 3D virtual planned and the achieved postoperative position for the three angles, the translation of the center of mass, and the implant position. Then a paired sample *t* test was performed to assess difference in achieved accuracy performing 3D-assisted correction osteotomies with conventional versus a patient-specific implants for all three angles and the translation. Finally, a sub-analysis regarding the accuracy of the correction was performed for cases in which small (case 1–5) or large (case 6–10) corrective osteotomies were performed. A calculated p value less than 0.05 was considered significantly different.

## Results

### Accuracy of corrective osteotomy

**Fig. 5:**
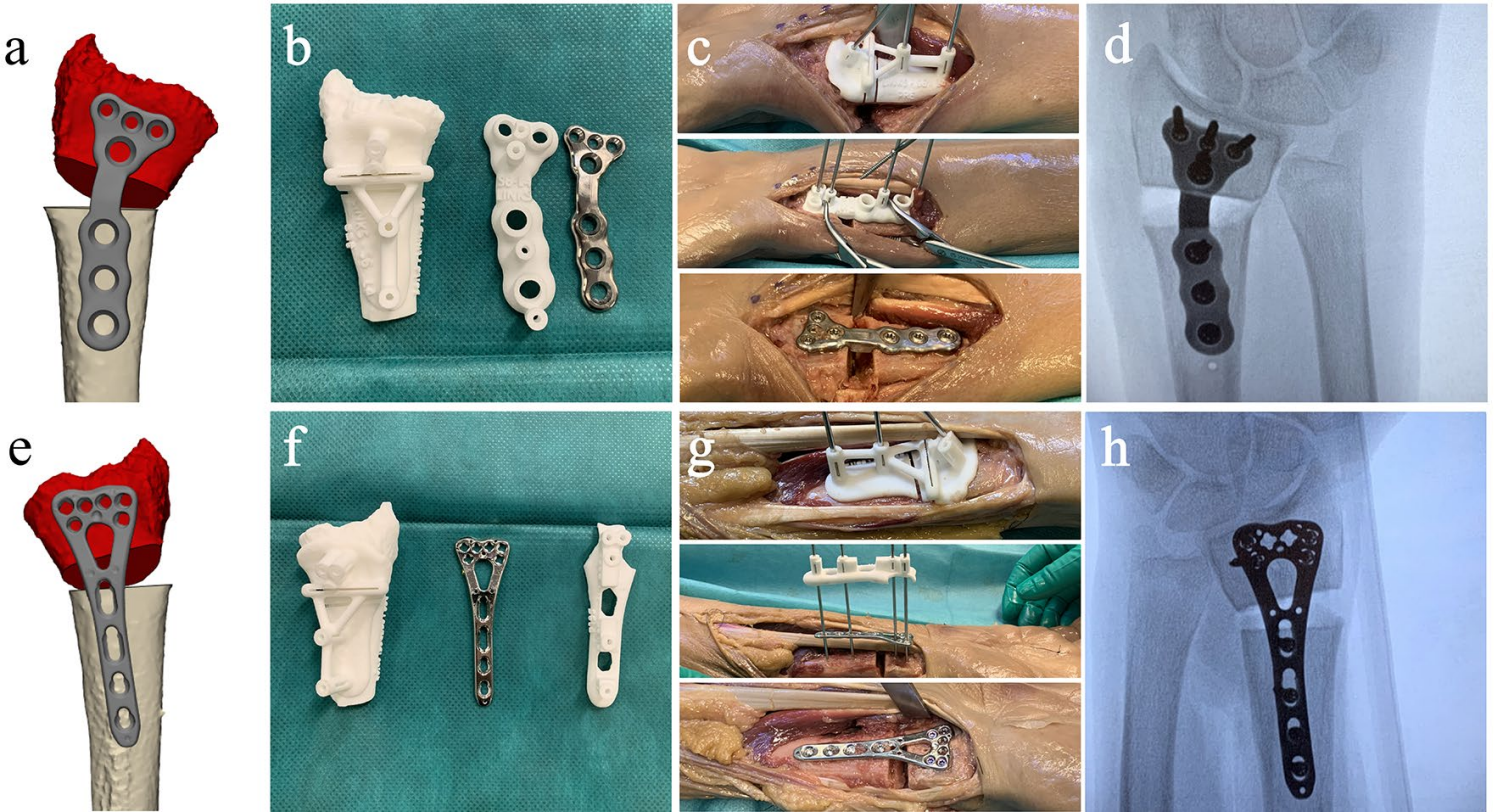
Surgical procedure of case 9 showing the left radius with a patient-specific implant (**a**–**d**) and the right radius with a pre-contoured conventional implant (**e**–**h**). From left to right: 3D model of the correction and implant (**a** and **e**). The milled titanium implant (**b**) or pre-contoured conventional implant (**f**) with their two corresponding guides. The perioperative photographs show the guides and implant in place (**c** and **g**) and the implant, screws, and corrections were verified by fluoroscopy before wound closure (**d** and **e**)

A total of 20 radii underwent 3D-assisted corrective osteotomies (Fig. 5). The median differences between the virtual 3D plan and the postoperative achieved position were 2.7° (IQR: 1.4–4.7) for all angles combined. The median differences for the separate angles were 2.6° (IQR: 1.6–3.9°), 1.4° (IQR: 0.6–2.9°), and 4.7° (IQR: 2.9–5.7°) for the rotation, dorsal, and radial angulation, respectively, across all 20 cases. The median difference between the center of mass of the distal radius between the 3D virtual plan and the achieved position was 2.4 mm (IQR: 1.3–2.9 mm).

The median differences between the 3D virtual plan and the postoperative achieved positions are displayed in Table 1. Radii treated with the patient-specific implants (all left radii) as compared to radii treated with pre-contoured conventional implants (all right radii) showed no significant differences in terms of rotation (*p* = 0.191), dorsal angulation (*p* = 0.522), radial angulation (*p* = 0.679), and translation of the center of mass (*p* = 0.933).

**Table 1:** Postoperative assessment of the performed corrective osteotomies

Side	Case	Rotation (°)	Dorsal angulation (°)	Radial angulation (°)	Translation (mm)
Left side (patient-specific implant)	Case 1	2.3	0.1	2.3	2.5
	Case 2	2.6	3.5	5.6	2.5
	Case 3	0.3	1.2	4.1	1.3
	Case 4	1.7	1.0	3.0	0.4
	Case 5	1.4	0.8	2.0	2.9
	Median (IQR) Small corrections	1.7 (1.4–2.3)	1.0 (0.8–1.2)	3.0 (2.6–4.1)	2.5 (1.3–2.5)
	Case 6	5.1	2.2	5.0	1.1
	Case 7	4.6	2.0	5.4	1.8
	Case 8	3.6	2.8	7.0	3.0
	Case 9	0.5	1.5	8.9	2.6
	Case 10	2.5	0.1	4.0	3.9
	Median (IQR) Larger corrections	3.6 (2.5–4.6)	2.0 (1.5–2.2)	5.4 (5.4–7.3)	2.8 (1.8–3.0)
Median overall		2.4 (1.4–3.4)	1.4 (0.9–2.1)	4.6 (3.3–5.6)	2.5 (1.4–2.8)
Right side (conventional implant)	Case 1	0.4	3.6	6.0	3.2
	Case 2	3.0	0.6	2.6	0.7
	Case 3	2.7	1.6	2.1	1.5
	Case 4	1.4	0.9	0.3	1.3
	Case 5	6.5	0.1	4.7	0.7
	Median (IQR) Small corrections	2.7 (1.4–3.0)	0.9 (0.6–1.6)	2.6 (2.1–4.7)	1.3 (0.7–1.5)
	Case 6	1.9	3.2	7.3	2.8
	Case 7	3.5	0.5	3.6	2.4
	Case 8	8.9	0.0	4.7	4.8
	Case 9	1.9	4.8	4.6	1.7
	Case 10	9.7	6.1	7.6	2.9
	Median (IQR) Larger corrections	3.5 (1.9–8.9)	2.0 (0.5–4.8)	4.7 (4.6–7.3)	2.8 (2.4–2.9)
Median (IQR)		2.9 (1.9–5.8)	1.3 (0.5–3.5)	4.7 (2.9–5.8)	2.0 (1.3–2.8)
*p* values Patient-specific versus conventional					
All corrections		0.191	0.522	0.679	0.933
Smaller corrections		0.398	0.985	0.829	0.533
Larger corrections		0.377	0.493	0.753	0.532

Accuracy was assessed in terms of rotation (degrees), dorsal angulation (degrees), radial angulation (degree), and translation (millimeters) between the 3D virtual surgical plan and the postoperative achieved position

### Accuracy of implant placement and screw trajectories

For the patient-specific implants, the translation of the implant (e.g., the median distance between the virtual planned position of the implant and the actual postoperative position) was 1.6 (IQR: 1.0–2.4) mm. For the conventional implant, the translation of the implant was 2.3 (IQR: 2.0–3.1) mm. No significant difference was found between the two groups with regard to all cases (*p* = 0.239), smaller corrections (*p* = 0.338) or larger corrections (*p* = 0.605).

For fixation of patient-specific implants, a total of 32 screws were placed using guided drill holes within the reposition guides. The median deviation in screw direction between the pre-planned trajectories and the postoperative result was only 4.0° (IQR: 1.9-4.9°). The screws for the conventional implants were not planned virtually; thus, no comparison can be made (Table 2).

**Table 2:** Positioning of implants as assessed by measuring the difference in millimeters between the center of mass of the virtually planned position and actual postoperative position of the implant

Side		Positioning of implants	Direction screws	
		(mm)	*N*	Median difference (°)
Left side (patient-specific implant)	Case 1	0.6	3	3.3
	Case 2	1.0	3	3.9
	Case 3	1.4	4	3.7
	Case 4	2.4	4	5.1
	Case 6	1.1	4	5.8
	Case 7	2.6	4	4.2
	Case 8	2.4	4	5.5
	Case 9	0.6	4	3.9
	Case 10	1.9	4	4.2
Median (IQR)		1.6 (1.0-2.4)	4.0	4.0 (3.9-4.9)
Right side (conventional implant)	Case 1	3.5		
	Case 2	2.2		
	Case 3	3.3		
	Case 4	0.4		
	Case 6	2.5		
	Case 7	2.0		
	Case 8	2.1		
	Case 9	1.6		
	Case 10	3.1		
Median (IQR)		2.3 (2.0–3.1)		

Direction of the screws (°) as assessed by measuring the angle in 3D between the virtually planned screw trajectory and the actually drilled direction postoperatively

## Discussion

Various companies offer virtual surgical planning services, including drill and saw guides, for performing 3D-assisted corrective osteotomies. Often, these products include a patient-specific implant. We tried to answer the question whether corrective osteotomies of the distal radius should be performed using a 3D work-up with pre-contoured conventional implants (i.e., of-the-shelf) or patient-specific implants (i.e., custom-made). This cadaveric study showed that both 3D technology workflows can be used to perform corrective osteotomies of the distal radius. Both types of implants can be used in combination with guides to facilitate the translation of the virtual surgical plan to the corrective osteotomy surgery of the distal radius. Both workflows have good postoperative results, the median postoperative angulation for both groups was less than 3° from the respective preoperative 3D plan, while the median translation difference of the distal radius bone segment was only 2.4 mm. This implies that the choice of implant should rely on other factors beyond just the accuracy of the correction, as discussed in the next sections.

3D-assisted corrective osteotomies are commonly used for treating radius malunions, often in combination with conventional implants [8–10, 23–31]. Nevertheless, CT-based postoperative evaluation of the accuracy of those corrections using 3D measurements is often lacking, which makes it challenging to compare our results with existing case series [8, 10, 23–27, 30]. Stockman et al. [12] reported on the feasibility of 3D printed osteotomy guides for performing corrective osteotomies of the distal radius. Their surgical approach involved multiple osteotomy guides which were also used to drill the reverse engineered holes for the fixation screws, whereas we used a slightly different surgical technique with an osteotomy as well as a reposition guide that fitted on top of the implant. Their preliminary results regarding the accuracy of the correction are promising (residual displacement of − 6 ± 6° for volar tilt and − 1 ± 5° for radial inclination) and in line with ours (1.4° (IQR: 0.6–2.9°) for volar tilt and 4.7° (IQR: 2.9–5.7°) for radial inclination). Corrective osteotomy surgery of the distal radius in combination with a patient-specific implant is reported in only a few studies [18, 19]. Dobbe et al. presented a case report on the application of a patient-specific distal radius locking plate [13], obtaining favorable outcomes (residual displacement of − 0.9°, 2.3°, and 1.7° for dorsal, radial, and rotational angles, respectively). Subsequently, Dobbe et al. [19] presented a case series involving corrective osteotomies of the distal radius treated with patient-specific implants. Their findings (residual displacement of 8.5° (7.9–9.5°) for rotation, 4.1° (3.3–7.7°) for radial angulation, and 2.8° (1.2–5.0°) for dorsal angulation) are consistent with our results for both the patient-specific implant as well as the pre-contoured conventional implant workflow. They concluded that the use of 3D technology in combination with patient-specific implants may improve the bone alignment and clinical outcome, as compared to the traditional approach based on 2D imaging modalities and conventional implants. Literature consistently states favorable clinical outcomes associated with 3D-assisted surgery for forearm osteotomies [7], irrespective of the implant type, which is in line with our observations. Our study adds to current knowledge because it provides a detailed technical description and direct comparison between patient-specific and pre-contoured conventional implants for corrective osteotomy surgery of distal radial malunions.

In the present study, the actual placement of implants deviated slightly from the preoperative virtually planned positions, with a median discrepancy of 1.6 mm (IQR: 1.0–2.4 mm) for patient-specific implants and 2.3 mm (IQR: 2.0–3.1 mm) for conventional implants. These minor deviations were considered clinically acceptable. Dobbe et al. [15] investigated the accuracy and reproducibility of 3D-assisted patient-tailored plates using artificial radii. They reported translation values of 1.2 ± 0.8 mm, which are consistent with those observed in our cadaveric study. However, their study did not consider soft tissue involvement, making their results less applicable to real surgical scenarios, whereas our study did take those into account. Omori et al. [16] investigated the use of patient-specific guides and implants for corrective osteotomy of the distal radius and humerus using human cadavers. They reported a translation error of less than 1.0 mm for implant placement, attributing it to limited surgical accessibility and the influence of surrounding soft tissues, which is in line with the results in our study. In our study, several screws were drilled in a pre-determined direction through screw holes in the reposition guides. The postoperative trajectories were compared to their preoperative plan and demonstrated only a median deviation of 4.0° (IQR: 3.9–4.9°), which is assumed to be acceptable for clinical use. These screws were placed within range of angles reported in previous literature, which includes 6.3 ± 3.4° [32] and 3.3° (IQR: 2.5–5.1°) [33] for tibial plateau fracture surgery, as well as 5.9° (IQR: 4–8°) [34] and 7.1° (IQR: 7–8°) [21] for acetabular fracture surgery. Overall, our study shows that 3D-assisted positioning of both pre-contoured conventional implants and patient-specific implants could be performed accurately within a few millimeters.

 Both fixation techniques, patient-specific implants and conventional implants, have advantages and disadvantages, which can influence the choice of implant. Our study examined the accuracy of using both implants; however, it is important to consider additional factors that should be explored in future research. Virtual surgery plans, surgical guides, and patient-specific implants are commercially available at a specific cost, but can also be developed in-house. For the in-house development of patient-specific implants, the workflow has to comply with the European Medical Device Regulatory (MDR) [35]. If conventional implants need to be pre-contoured by the surgeon, a 3D printed model is required, necessitating the availability of all these facilities within the hospital. Implementing these innovative workflows in-house necessitates substantial resources, a dedicated team, validated software packages, and production facilities, which entails significant financial and time investments. Second, patient-specific implants can serve as a mold for the planned correction, providing the surgeon with direct visual feedback and confidence during the surgical procedure. Nevertheless, a drawback of this approach is the limited flexibility for the surgeon to deviate from the preoperative surgical plan if unexpected changes occur during surgery. Lastly, it is important to note that malunions often coincide with abnormal bone growth; thus, conventional “anatomical” implants may not be capable of facilitating proper alignment due to the complexities of the underlying deformed bone structures. This highlights a potential advantage of patient-specific implants, as they are presumed to overcome challenges associated with abnormal bone growth. All elements mentioned above should, therefore, be taken into consideration at the start of a 3D-assisted workflow. If preoperative virtual 3D analysis indicates that pre-contouring is needed and feasible with the underlying bone structure and required degrees of correction, and all facilities are available, this can be a good clinical option for correction of the distal radius. However, if the preoperative plan indicates a preferred choice for patient-specific plan due to complex anatomy of the malunited bone (e.g., irregular bony surface due to fracture healing and exostosis), or the 3D workflow for pre-contouring is not available, it is recommended to opt for a patient-specific implant.

It is important to acknowledge that this cadaveric study is not without limitations. First, the study employed reverse deformities, including translations and rotations, to simulate osteotomy surgeries. However, this was the only possible way to simulate corrective osteotomy surgery in a human cadaveric study, since none of the available human specimens had deformities. The planned corrective surgeries in this study were based on smooth bone surfaces, assuming a simplified bone–implant interface. In reality, the presence of abnormal bone growth, irregular surfaces due to pervious fractures, may interfere with the performance and fit of the conventional implants. Second, this study focused solely on 3D-assisted surgery and did not compare results with corrective osteotomies based on conventional 2D imaging modalities (e.g., without virtual surgical planning, 3D printed models and guides). We are aware that in many centers, corrective osteotomy surgeries are still performed using only 2D imaging modalities, while literature shows 3D-assisted corrective osteotomy surgery seems to improve patient-reported outcomes and reduces complications compared to conventional 2D approaches [7]. However, a comparison between 2D and 3D techniques was not in the scope of this study. Finally, it is important to note that this study does not include an analysis of costs. The workflows of both techniques involve various steps that include expenses related to software, equipment, materials, and personnel. The magnitude of these costs depends on factors such as the availability of in-house resources and the geographical location of the implementation. Therefore, future research perspectives should consider conducting a comprehensive cost-effectiveness study comparing the two techniques.

In conclusion, this cadaveric study aimed to evaluate the accuracy of the 3D-assisted corrective osteotomy of the distal radius utilizing either a patient-specific (i.e., custom-made) implant or a pre-contoured conventional implant (i.e., of-the-shelf). This study shows that a 3D-assisted workflow with a virtual surgical planning in combination with both an osteotomy guide and a reposition guide facilitates precise corrective osteotomy of the radius. Our 3D-assisted workflows effectively translated the virtual preoperative plan into the surgical procedure. The surgical procedure could be performed with either a pre-contoured conventional implant or patient-specific implant, and screws could be placed accurately using the drilling holes in the reposition guides. So, other factors such as presence of in-house facilities, the ability to properly pre-contour the conventional implant, the condition of the underlying bone structure and available expertise, should contribute to the decision-making process for selecting the appropriate implant.

## Appendix I

The degree of malposition of the distal radius for each case that required correction through reversed correction osteotomies.

**Table:**

Side	Case	Rotation (°)	Dorsal angulation (°)	Radial angulation (°)	Translation (mm)
Left side (patient-specific implant)	Case 1	9.3	10.3	9.4	6.6
	Case 2	8.4	12.0	8.1	4.8
	Case 3	6.6	9.7	6.4	4.9
	Case 4	10.0	12.7	5.3	5.0
	Case 5	12.8	10.3	8.4	3.8
	Case 6	23.4	29.8	12.7	7.9
	Case 7	4.6	25.3	22.9	8.3
	Case 8	26.6	28.0	3.8	6.3
	Case 9	16.1	28.0	18.5	8.9
	Case 10	20.5	20.8	13.9	5.7
Right side (conventional implant)	Case 1	8.2	11.5	10.5	6.8
	Case 2	4.2	8.4	9.1	4.0
	Case 3	6.0	9.0	6.8	4.4
	Case 4	15.3	10.0	5.0	5.0
	Case 5	11.8	9.9	8.0	3.1
	Case 6	23.3	29.7	13.8	7.4
	Case 7	7.0	28.2	16.1	7.6
	Case 8	25.0	28.0	0.7	6.7
	Case 9	19.1	28.9	15.2	8.2
	Case 10	20.7	18.8	15.4	6.6
